# Enhancing saccharification of wheat straw by mixing enzymes from genetically-modified *Trichoderma reesei* and *Aspergillus niger*

**DOI:** 10.1007/s10529-015-1951-9

**Published:** 2015-09-09

**Authors:** Yanping Jiang, Alexandra Vivas Duarte, Joost van den Brink, Ad Wiebenga, Gen Zou, Chengshu Wang, Ronald P. de Vries, Zhihua Zhou, Isabelle Benoit

**Affiliations:** Key Laboratory of Synthetic Biology, Institute of Plant Physiology and Ecology, Shanghai Institutes for Biological Sciences, Chinese Academy of Sciences, Shanghai, 200032 China; CBS Fungal Biodiversity Centre, Uppsalalaan 8, 3584 CT Utrecht, The Netherlands; Fungal Molecular Physiology, Utrecht University, Uppsalalaan 8, 3584 CT Utrecht, The Netherlands; Key Laboratory of Insect Developmental and Evolutionary Biology, Institute of Plant Physiology and Ecology, Shanghai Institutes for Biological Sciences, Chinese Academy of Sciences, Shanghai, 200032 China

**Keywords:** *Aspergillus niger*, Genetically-modified strains, Saccharification, *Trichoderma reesei*, Wheat straw

## Abstract

**Objectives:**

To increase the efficiency of enzymatic hydrolysis for plant biomass conversion into renewable biofuel and chemicals.

**Results:**

By overexpressing the point mutation A824 V transcriptional activator Xyr1 in *Trichoderma reesei*, carboxymethyl cellulase, cellobiosidase and β-d-glucosidase activities of the best mutant were increased from 1.8 IU/ml, 0.1 IU/ml and 0.05 IU/ml to 4.8 IU/ml, 0.4 IU/ml and 0.3 IU/ml, respectively. The sugar yield of wheat straw saccharification by combining enzymes from this mutant and the *Aspergillus niger* genetically modified strain Δ*creA*/*xlnR*_c_/*araR*_c_ was improved up to 7.5 mg/ml, a 229 % increase compared to the combination of wild type strains.

**Conclusions:**

Mixing enzymes from *T. reesei* and *A. niger* combined with the genetic modification of transcription factors is a promising strategy to increase saccharification efficiency.

**Electronic supplementary material:**

The online version of this article (doi:10.1007/s10529-015-1951-9) contains supplementary material, which is available to authorized users.

## Introduction

Depleting fossil energy sources and related environmental issues have stimulated great interest in alternative renewable biofuel sources (Nigam and Singh 2011). Plant biomass is a promising source of raw material for biofuel production (Klemm et al. 2005). A key bottleneck for the bioconversion of plant biomass into biofuels is the high cost of enzymes and their insufficient efficiency in releasing sugars from lignocellulose (Falkoski et al. 2012). To achieve cost-effective bioconversions, maximizing the enzyme yield and optimizing enzyme mixtures is considered an attractive approach with great potential (Pal and Chakraborty 2013).

*Trichoderma reesei* and *Aspergillus niger* are established industrial producers of enzymes that degrade plant biomass (Dos Santos Castro et al. 2014). The main activators of cellulolytic and xylanolytic gene expression in *T. reesei* and *A. niger* are Xyr1 and XlnR, respectively (Stricker et al. 2006) (van Peij et al. 1998). Constitutive expression of Xyr1 in *T. reesei* leads to a significant elevation of the production of the main biomass degrading enzymes (Mach-Aigner et al. 2008). The mutation A824V of Xyr1 improves xylanase and basal cellulase gene expression in *T. reesei* (Derntl et al. 2013). In *A. niger,* constitutive activation of XlnR also results in constant xylanase production even under repressing conditions (Hasper et al. 2004). *A. niger* contains a second hemicellulolytic transcriptional activator, AraR, which has sequence homology to XlnR but is only present in the Eurotiales (Battaglia et al. 2011). This regulator controls the production of extracellular L-arabinose releasing enzymes in *A. niger*.

Although *T. reesei* produces an efficient set of cellulolytic enzymes, β-d-glucosidase production is low, while the opposite was reported for *A. niger* (van den Brink and de Vries 2011). Therefore, plant biomass hydrolysis can be improved by using *T. reesei* cellulase complemented with enzymes from other organism (Tabka et al. 2006). In this study, improvement of wheat straw saccharification was achieved by overexpressing a constitutive version of *xyr1* in *T. reesei* and combining the enzymes produced by this strain with those from an *A. niger* strain in which *creA* was deleted and constitutive versions of *araR* and *xlnR* were inserted.

## Materials and methods

### Microbial strains, plasmids and cultivation

*Escherichia coli* DH5α was used for cloning. *Agrobacterium tumefaciens* AGL-1 served as a T-DNA donor for *T. reesei* transformation. All fungal strains are listed in Supplementary Table 1. A uridine auxotrophic *ura5*- negative strain 2-3 was used for overexpression of native or mutated A824V *xyr1*. A T-DNA binary vector, Npxbthg, containing the selecting marker gene *ura5* coding for orotate phosphoribosyltransferase from *Penicillium oxalicum* under control of the *Aspergillus nidulansgpdA* promoter and *trpC* terminator, was used to construct the transformation vectors for *T. reesei*.

Transformants of *T. reesei* were selected as described previously (Gruber et al. 1990), with cefotaxime (300 μM) and 0.2 % Triton X-100 (v/v). Each positive transformant was used to generate genetically stable and correct mono-conidia, which were confirmed by PCR. All *Aspergillus* strains were cultivated in *Aspergillus* minimal medium (de Vries et al. 2004). Wheat straw, xylan from beechwood, Avicel, glucose and xylose were used as carbon sources. An *A. niger* strain with a *creA* deletion was used (van den Brink et al. 2014).

The expression of *araR* and *xlnR* was made constitutive by deleting the *C*-terminus from Pro646 and Leu668, respectively (Hasper et al. 2004). The *A. niger* Δ*cre*A background was used in both case.

### Overexpression of native or constitutive *T. reeseixyr1*

The native and mutated A824V *xyr1* PCR fragment were amplified from genomic DNA of QM9414 by using primers in Supplementary Table 2 and cloned between the *pdc* promoter and *trpC* terminator into the *Bam*HI site of Npxbthg using the ClonExpress MultiS kit (Vazyme Biotech) resulting in vector Npxbthg-OEXyr1 and Npxbthg-OEmXyr1. These vectors were transformed into the recipient strain using *A. tumefaciens*-mediated fungal transformation.

### Cellulase activity, SDS-PAGE and silver staining

Activity of carboxymethyl cellulase (CMCase), cellobiosidase (*p*NPCase) and β-d-glucosidase (*p*NPGase) were assayed as described previously (Ma et al. 2011). Significance analysis was performed by Student’s *t*-test using a two-tailed test. SDS-PAGE (12 %, w/v) with silver staining was performed to analyze extracellular protein profiles. Due to interference of wheat straw with the usual protein determination methods, the total protein determination was estimated by visual inspection of the SDS-PAGE loaded with 20 μl of samples.

### Saccharification of wheat straw

Saccharification was performed in 10 ml containing 2 ml enzyme mixtures, 8 ml saline/sodium acetate buffer (pH 5, 50 mM) and 0.3 g washed wheat straw, and incubated at 30 °C and 200 rpm. The ratio of the enzyme mixtures from *T. reesei* and *A. niger* was 1:1 taken from wheat straw liquid (3 %, w/v) after 4 days of cultivation and filtered through a 0.22 µm nylon filter (van den Brink et al. 2014). For control measurements, 2 ml saline/sodium acetate buffer (pH 5, 50 mM) substituted the enzyme mixtures. The reducing sugar yield was quantified daily using the DNS method (Ma et al. 2011). HPLC was used to determine individual sugar concentrations. Sugar analysis was performed on a Thermo Scientific 5000 + HPLC-PAD system. A multi-step gradient was used to analyze the sugars. A flow rate of 0.3 ml/min was used on a CarboPac PA1 column. Guard column: Dionex CarboPac PA1 BioLC 2 × 50 mm. Main column: Dionex CarboPac PA1 BioLC 2 × 250 mm. A pre flow of 18 mM NaOH was used before injection to equilibrate the column. During a total running time of 50 min, the following solutions were used: A, water; B, 100 mM NaOH; C, 100 mM NaOH with 1 M sodium acetate. 18 % B was used for the first 20 min followed by a 10 min linear gradient to 40 % C, 0 % B. 100 % C for 5 min. To rinse the acetate, 100 % B was used for 5 min. 10 min 18 % B was used for 10 min to rinse the column. Quantification was performed by external standard calibration. Reference sugars (Sigma-Aldrich) were used from 2.5 to 200 µM. The data obtained are the results of two independent biological replicates and for each replicate three technical replicates were assayed.

## Results and discussion

### Overexpression of native and constitutive *xyr1* leads to significant increase of enzyme yields

**Fig. 1:**
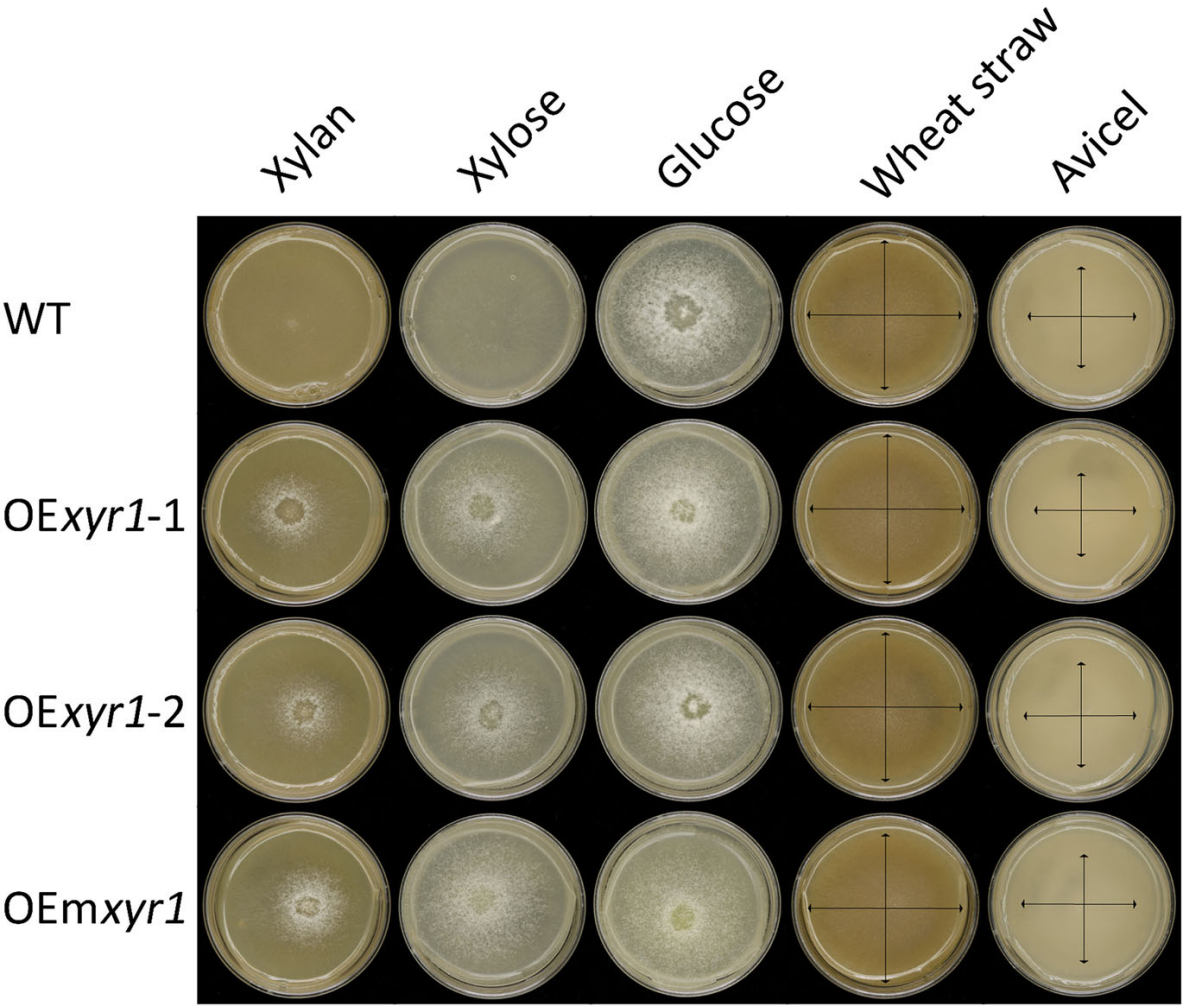
Growth profiles of the parental strain (WT), QM9414 uridine auxotrophic mutant; OE*xyr1*-1 and OE*xyr1*-2, overexpressed *xyr1*; OEm*xyr1*, overexpressed constitutive *xyr1* (A804V). 1000 spores in 2 µl ACES were spotted on the center of minimal medium plates with 1 % carbon source (W/V, xylan from beechwood, xylose, glucose, wheat straw and Avicel, respectively) and incubated at 30 °C. Pictures were taken three days (3d) after inoculation. The arrows show the edges of the colonies

The three *T. reesei* strains: wild type, overexpressed *xyr1* and overexpressed constitutive *xyr1*, were grown on minimal medium with 1 % carbon source (xylan, xylose, glucose, wheat straw and Avicel). No significant growth improvement between the *T. reesei* wild type and Xyr1 mutant strains was observed on glucose, Avicel or wheat straw (Fig. 1). In contrast, on xylan and xylose, the colony size of the Xyr1 mutants was considerably increased compared to the wild type. This correlates with the previously demonstrated role of Xyr1 in xylan and xylose utilization (Stricker et al. 2006). Furthermore, overexpression of the constitutive *xyr1* resulted in better growth than that of native *xyr1*, suggesting the point mutation (A824V) in the gene encoding Xyr1 enhances the utilization ability of xylan and xylose by *T. reesei*.

**Fig. 2:**
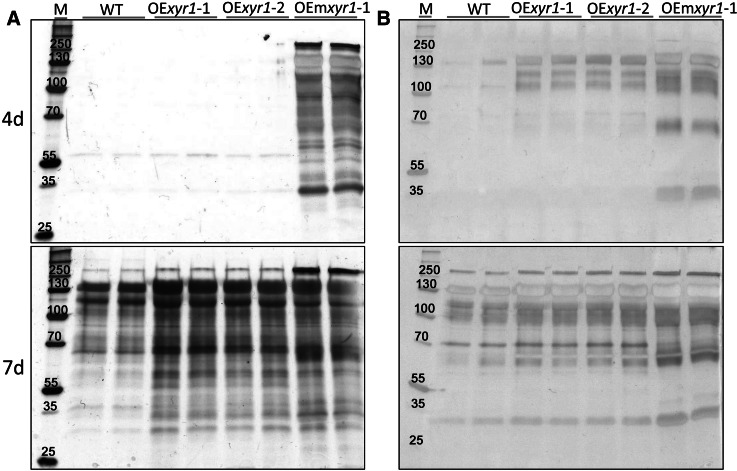
Exo-protein profiles of the wild type and mutant strains grown on Avicel and wheat straw. The strains were inoculated at 10^6^ spores/ml into 250 ml flasks containing 50 ml minimal medium with **a** Avicel PH-101(1 %, W/V) or **b** wheat straw (3 %, W/V) and cultivated at 30 °C, 200 rpm. Culture supernatants of the fungal strains were collected after four (4d) and seven (7d) days by centrifugation at 4 °C and 12,000×*g* for 10 min

Cellulase activities of *T. reesei* wild type and the native or constitutive *xyr1* transformants OE*xyr1*-1, OE*xyr1*-2, OEm*xyr1* were measured in supernatants from Avicel and wheat straw cultures. All three strains produced a larger amount of secreted proteins than the wild type (Fig. 2). This result contrasts with the overexpression of XlnR in *Aspergillus vadensis* where additional copies of the transcriptional activator had no effect on protein production (Bouzid and de Vries 2014). OEm*xyr1* produced the highest protein level and showed the earliest protein production, particularly in Avicel (Fig. 2a). The other two mutants showed an earlier secretion of proteins with wheat straw than with Avicel (Fig. 2b). This may be linked to a higher fungal biomass formation as suggested by their growth profiles (Fig. 1).

**Fig. 3:**
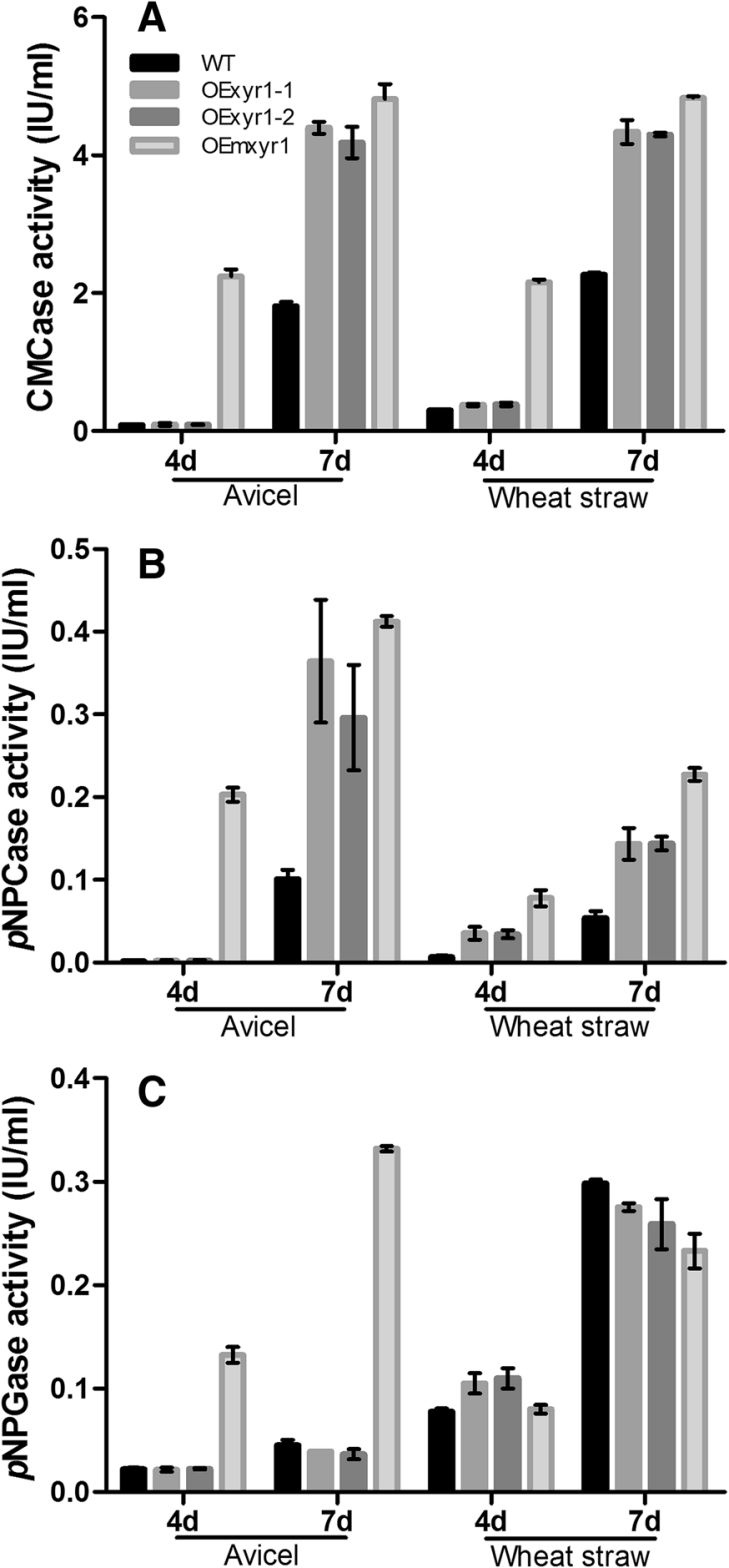
Cellulase activities measured from the wild type and the mutant strains grown on Avicel and wheat straw. **a** CMCase, **b** *p*NPCase and **c** *p*NPGase activities. The culture condition and the sample treatment were the same as for Fig. 1. *Vertical bars* indicate standard deviation of biological duplicates

Consistent with extracellular protein production, cellulase activity of the mutant strains was considerably improved compared to the wild type, in agreement with earlier reports (Mach-Aigner et al. 2008; Derntl et al. 2013). Interestingly, the overexpressed constitutive mutant OEm*xyr1*, had an even greater effect. After 4 days of growth, both in Avicel and wheat straw, only OEm*xyr1* produced high cellulase activity. Moreover, after 7 days’ growth on Avicel, OEm*xyr1* showed increased CMCase, *p*NPCase and *p*NPGase activities from 1.8 IU/ml, 0.1 IU/ml and 0.05 IU/ml to 4.8 IU/ml, 0.4 IU/ml and 0.3 IU/ml, respectively, (Fig. 3a–c). No significant differences were observed for the *p*NPGase in wheat straw medium, but OEmxyr1 produced more of this activity in Avicel medium (Fig. 3c), demonstrating the influence of the carbon source on the regulation of the β-d-glucosidase in the constitutive Xyr1 strain (Castro et al. 2014).

### Enhancing wheat straw saccharification

**Fig. 4:**
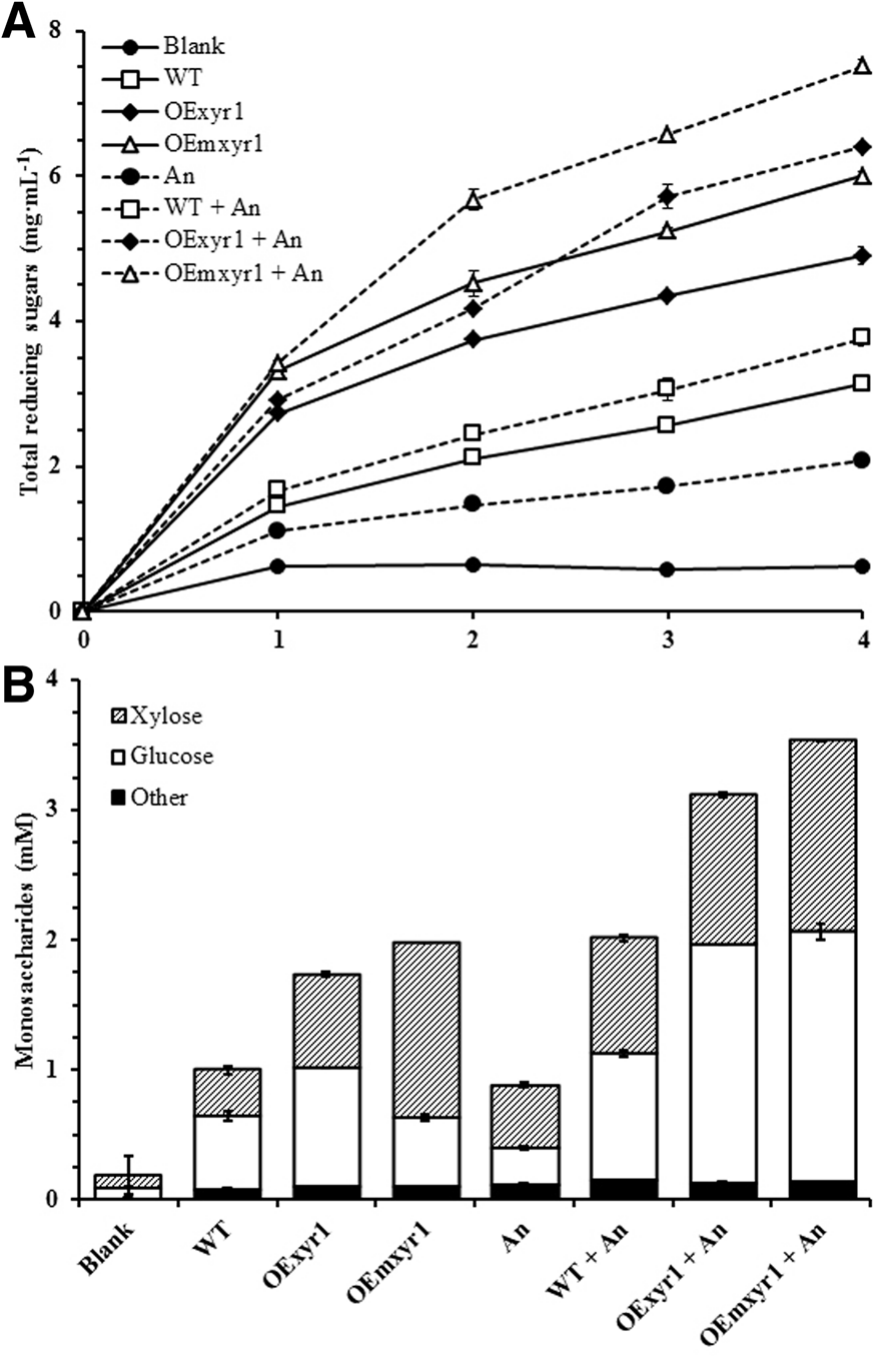
Saccharification analysis of wheat straw. **a** Total reducing sugar yield; **b** Individual sugar concentrations. Wheat straw was filtered, autoclaved in demi-water and washed thoroughly with demi-water to remove the soluble sugars. *Error bars* indicate standard deviation of biological duplicates

To evaluate the effect of the overexpressed constitutive Xyr1, saccharification experiments were performed on wheat straw using supernatants of wheat straw-grown cultures. *T. reesei* OE*xyr1* and OEm*xyr1* showed higher release of sugar compared to the reference strain (Fig. 4). The OEmxyr1 released by far the largest amount of sugar which is in line with the measured activities. Interestingly, this increased saccharification capacity is mainly due to xylose release and not due to release of glucose (Fig. 4b).

A triple mutant from *Aspergillus niger* was further added to the experiment. This triple mutant is a double constitutive *araR* and *xlnR* made from a Δ*creA* background (Δ*creA*/*xlnR*_c_/*araR*_c_).

Combining the enzyme mixtures of these strains with the enzyme mixtures of the *T. reesei* wild type significantly improved saccharification. Combining enzyme mixtures of OE*xyr1* and OEm*xyr1* with *A. niger* Δ*creA*/*xlnR*_c_/*araR*_c_ resulted in even better wheat straw saccharification and the best sugar yield (7.51 mg/ml) was achieved by combining enzymes from OEm*xyr1* and mutant Δ*creA*/*xlnR*_c_/*araR*_c_. This is 229 % of the combination of wild type strains (Fig. 4), which goes beyond results obtained previously (van den Brink et al. 2014).

**In conclusion**, we significantly increased the saccharification of wheat straw by combining the enzyme mixtures of two engineered strains: *T. reesei* OEm*xyr1* and *A. niger* Δ*creA*/*xlnR*_c_/*araR*_c_.

## Electronic supplementary material

Supplementary material 1 (DOCX 14 kb)
