# Supplementary material for: Enhancing saccharification of wheat straw by mixing enzymes from genetically-modified Trichoderma reesei and Aspergillus niger
Source: Biotechnol Lett. 2015 Sep 9;38:65–70. doi: 10.1007/s10529-015-1951-9 (PMC4706842; doi:10.1007/s10529-015-1951-9)
Supplement: Supplementary file 1 — Supplementary material 1 (DOCX 14 kb) [file 10529_2015_1951_MOESM1_ESM.docx]

**Supporting information**

Supplementary Table 1. List of *T. reesei* and *A. niger* strains.

| Species | Strain name | Description | Reference |
| --- | --- | --- | --- |
| *T. reesei* | WT | A uridine auxotrophic *ura5^-^* negative strain 2-3 derived from QM9414 | This study |
|  | OE*xyr1* | Constitutive overexpression of native *xyr1* in WT | This study |
|  | OEm*xyr1* | Constitutive overexpression of mutanted *xyr1* in WT | This study |
| *A. niger* | Δ*creA* | *creA* disruption strain | (van den Brink et al. 2014) |
|  | Δ*creA*/*xlnR*_c_/*araR*_c_ | Constitutive overexpression of *xlnR* and *araA* in *creA* disrupted strain | Vivas-Duarte and de Vries, in preparation |

Supplementary Table 2. List of primers.

| Primers | Sequences (5 '–3 ')_a_ |
| --- | --- |
| Ppdc-F | CAGGTCGACTCTAGAGGATCCATGAAAGGAGGGAGCATTCTTCGAC |
| Ppdc-R | GATTGTGCTGTAGCTGCGCTGCTTT |
| Native *xyr1*-F | AGCGCAGCTACAGCACAATCATGTTGTCCAATCCTCTCCGTC |
| Native *xyr1*-R | TTAGAGGGCCAGACCGGTTC |
| Mutated *xyr1*-F | GGTATCGGCTGTCGAAGCTATTAGC |
| Mutated *xyr1*-R | GCTAATAGCTTCGACAGCCGATACC |
| Ttrpc-F | GAACCGGTCTGGCCCTCTAAAGTAGATGCCGACCGGATC |
| Ttrpc-R | GCTTAACTATGCGGCGGATCCCAGGGCTGGTGACGGAATTT |

_a_ Restriction sites and mutated DNA sequences are underlined
